# The characteristics of organized sport and physical activity initiatives for older adults in Sweden

**DOI:** 10.3389/fspor.2023.1168312

**Published:** 2023-09-04

**Authors:** Helena Ericson, Susanna Geidne

**Affiliations:** Division of Sport Science, School of Health Sciences, Faculty of Medicine and Health, Örebro University, Örebro, Sweden

**Keywords:** older adults, sports, salutogenic, settings-based, drop-out, recruiting

## Abstract

There is a common understanding that sport and physical activity can be important to address healthy aging. There are individual-level recommendations about how much physical activity people of different ages should engage in to gain health benefits, but at the same time there are no recommendations for how organizations should organize physical activities to suit as many people as possible for as long as possible. The purpose of this study was to explore the characteristics of sport and physical activity initiatives that older adults participate in. Different ongoing sport and physical activity initiatives that involve older adults were investigated regarding their focus, organization, intensity and organizer, and in relation to their costs, booking opportunities and recruitment. The study was conducted with a cross-sectional design using the Salutogenic Physical Activity Health Resources Questionnaire (SPAHRQ). The study included 27 different initiatives with 372 participants (60% women) ranging from 60 to 96 years of age. A health-promoting, salutogenic settings-based approach, and specifically the concepts drop-in, drop-through and drop-over were used in discussing recommendations for the organization of sport and physical activity for older adults. The main findings were that who organizes the sport and physical activity initiative seems to affect the characteristics of *how* it is organized and *what characterizes* the participants in it. Despite the differing characters of sport and physical activity initiatives, the majority of older adults are recruited by internal contacts like friends and family. Which older adults participate in which initiatives is explained mostly by the age and gender of the participants. In conclusion, to attract as many older adults as possible, organizations should work with lowering the thresholds, as well as within and between, organizations, and raise the threshold for dropping out of sports and physical activity.

## Introduction

There is a widespread belief that sport and physical activity is an important “player” with regards to addressing healthy aging. There are individual-level recommendations about how much physical activity people of different ages should engage in to gain health benefits (1), but at the same time there are no recommendations for how organizations should organize physical activities to suit as many people as possible for as long as possible. The purpose of this study is thus to explore the characteristics of sport and physical activity initiatives that older adults participate in and to discuss this in relation to recommendations for how arrangers could organize activities for older adults.

### Participation in physical activity and sport for older adults

Participation in organized sport and physical activity provides substantial physical, psychological and social health benefits for older adults (2–4). Regardless of these apparent benefits, older adults represent one of the least physically active groups in society across the globe (5, 6).

At the same time, few older adults participate in sport clubs (7, 8). Most older adults instead engage in physical activity in their own regime, the most common activity being walking (9). Older adults also have different purposes, presumptions, and background regarding sport and physical activities in organized form (10, 11) and having a history of engaging in sport and physical activity throughout life can lead to being generally more active in older age (12). It can thus be concluded that older adults are a heterogeneous group with regard to sport and physical activity (10, 13), although what they find meaningful in the activities can be quite similar (14).

### Organization of sport and physical activity for older adults

Older adults have mainly been explored regarding individual perspectives on engaging in physical activity, for example motivation, confidence and resilience (15, 16) as well as barriers to participating (10, 17, 18) and reasons for dropping-out of organized sport (19).

There are limited studies, however, about the organizational perspective on older adults and sport (20), and more attention could be paid to these dimensions of the “how” in organizational contexts and contents (4). The studies that have focused on the organizational dimensions of sport for older adults emphasize aspects such as adaptations to create age-appropriate opportunities (19), modifications in rules (21), or even creating new forms of sport, similar to walking soccer (22). Some studies conclude that older adults often are recruited to sport by internal contacts, like personal invitations (21, 23), and that external recruiting is important but needs more explicit strategies, for example highlighting the different experiences and health and social benefits that participation can give (21). Previous studies are highlighting that senior associations are emphasized as an important place of recruiting and communicating with old adults about PA (21, 23, 24). Participation fees are mentioned as a structural feature that can affect organizing perspectives; for example, passing over used equipment between participants could be a solution for older adults with different economic possibilities (21). So, with this in mind, lowering the thresholds, for example meaning it should be easy to start exercise without no earlier knowledge or experience and where recruiting also is especially important for increasing diversity and reducing social inequality (24).

### The sport and physical activity landscape for older adults in Sweden

There is not much research on what physical activities older adults participate in globally, and this is also the case for Sweden. One smaller Swedish survey revealed that less than one third of the older adults in Sweden were members of a sports club, and slightly more men than women (9). In a national survey on older adults participating in sports clubs it was concluded that this group was homogenous related to socio-economic status (with a relatively high socio-economic status), but heterogeneous in relation to which sport and physical activity they engage in (25).

In a study from Huffman and Amireault (13) they argue that the organizing of activities for older adults needs to consider a multilevel perspective, such as a socio-ecological framework. This is a conclusion that studies on youth sports have also reached (26, 27). However, there has been a limited focus on the organizational perspective in research on older adults' sports participation. Studying sport and physical activity initiatives that are ongoing and exploring how they are designed can thus be a way to understand older adults' participation in sport and physical activity in later life. If they are organized traditionally and similarly for all age groups and genders, there is a danger of missing out on possible participants and perhaps creating overly narrow or conservative activities which could lead to limitations in encouraging participation (13). This research therefore aims to identify what characterizes (in this particular study: costs, recruitment, booking system, type of activity) sport and organized physical activity initiatives for older adults.

### The present study

The purpose of this study is to explore the characteristics of sport and physical activity initiatives in Sweden that older adults participate in. In the study, we will investigate different ongoing sport and physical activity initiatives that involve older adults with regard to their focus, organization, intensity, and organizer (see Table 1) (28). We will answer the following research questions:

1) What characterizes the sport and organized PA initiatives that older adults participate in?
2) What characterizes older adults who participate in different sport and organized PA initiatives?

**Table 1:** Activity categories with inclusion criteria.

Activity categories	Inclusion criteria
Activity organizer	Activity organizer refers to the organizing club or association. A senior association is one where activities take place only for older adults (60+). Sport clubs can have both senior-specific initiatives and activities for other age groups and different purposes; the participants included in this study are in senior activities only.
Activity focus	Activity focus, with the possibility to compete or participate exclusively as a leisure activity. Here activities are categorized as either/or; i.e. if there is a possibility to compete (ex. golf, tennis) even it is categorized as competition, even if someone chooses not to do so.
Activity organization	Activity organization concerns activities requiring a team (or at least an opponent to play against) to take part in, as opposed to individually performed activities (though still in a group).
Activity intensity	Activity intensity describes the level of intensity of the activity and was labelled as low intensity (MET < 4) or medium-to-high intensity (MET > 5) according to the MET table scale (28).

We will investigate this using four proposed activity categories, focus, organization, intensity, and organizer, analysing these in relation to both organizational variables and participant variables to answer the two research questions. In this way, questions like these can give a more complete understanding of the characteristics of organized sport and physical activities for older adults.

In this study, we have used a salutogenic settings-based approach to health promotion in line with (26) to discuss our results in relation to recommendations for how organizers could organize activities for older adults.

The salutogenic settings-based approach has its origin in both salutogenic theory Antonovsky (29, 30) applied to a sport context (31) and a health-promoting, settings-based approach for sport clubs Geidne et al. (32), and Van Hoye et al. (33). The salutogenic perspective in health promotion stems from a critique of a dichotomous classification of people as healthy or diseased, and a focus on disease “as the departure from the norm and the normal, as that which has to be explained” (30). In this study we will specifically use the concepts employed in (26), i.e., drop-in, drop-through and drop-over, in discussing recommendations for the organization of sport and physical activity for older adults. These concepts are also discussed in relation to each other using the word thresholds which can be thought of as steps/changes/phases more than clear cut-points, to discuss how to lower the thresholds into the activities, within the organizations and between the organizations to increase the threshold of drop-out of PA and sport initiatives.

## Materials and methods

### Study design and context

This study was conducted with a cross-sectional design using a questionnaire to collect data from 27 ongoing organized sport and physical activity initiatives with older adults in Sweden. The 27 initiatives were either organised by a senior association or a sports club (none in private regime). Ethical approval was granted by the Swedish Ethical Review Authority (Dnr 2019-04818). The Swedish sport movement is traditionally organized as non-governmental organisations but has extensive government funding that could be described as an “implicit contract” (34). In recent decades there has also been an increase in other types of actors, like companies and other organizations that run sport activities in Sweden (35), a trend that can also be observed in other western countries (36).

### Participants and procedure

The sport and physical activity initiatives were located among both senior associations and sport clubs with ongoing regular sport and physical activities in and around a medium-sized city. The sampling was conducted with a snowballing strategy (37) (using network contacts in the county sports federation, organizations, and others) to find as many of the ongoing sport and physical activity initiatives in the region as possible. The criteria for inclusion were that sport and physical activity initiatives should be organized and offer weekly group activities for participants 60 years and above. Exclusion criteria were not organizing for a group or being explicitly organized as rehabilitation, prevention, or treatment of disease, for example an activity prescribed or recommended by the health care system.

The recruitment process consisted of contacting the head of the organization/sport club manager by phone. The participants in the 27 recruited initiatives were initially given oral information by the group leader at one of their regular sessions, one week before the data collection took place. Written and oral information were then provided to the participants and informed written consent was received. The questionnaires were handed out in person (in paper and pen form) by the first author. Reading glasses were available, in case participants did not bring them to the training sessions. The participants could fill out the survey on site or request a postage-paid, addressed envelope to send it from home. It was possible to ask questions while filling out the questionnaire on site and also to get in contact with the first author by e-mail or phone. All participants were active in these ongoing organized sport and physical activity initiatives on a voluntary basis. The study took place from December 2019 to early March 2020, when the data collection had to cease due to the Covid-19 outbreak in early spring 2020. All questionnaires were coded and handled confidentially.

### Measures

The questionnaire that was used is the Salutogenic Physical Activity Health Resources Questionnaire (SPAHRQ), developed from previous studies on physically active older adults (14, 38). SPAHRQ was developed using a salutogenic theory-driven approach as introduced by Antonovsky (30, 39) and further developed for use in studies within sport and physical education (31, 40). The SPAHRQ questionnaire has been used to report other outcomes in the same sample but in this particular study variables focusing sport and PA participation are presented. The questionnaire has been used in an earlier study (14). Briefly, salutogenic theory assumes that how we understand health is closely related to both our social, cultural, and natural environment, and our physical, social, and mental resources as individuals (31, 41). A salutogenic perspective consequently helps us consider individual and organizational aspects of older adults' participation in organized sport and physical activity initiatives as well as the relation between individual and organizational aspects. The focus of this study is on organizational-level characteristics of the physical activity initiative in which the older adults participate.

As a tool for analysis, the initiatives were first categorized by the authors into four dichotomous pairs inspired by Eime and colleagues' (42) categorization of leisure-time PA by type (which sport or exercise), mode (team-sport, individual sport, exercise and non-organized PA) and setting (school, sport club, home, public park, etc.). Since these terms are based on adolescents' leisure-time PA, we have adapted and refined them into the four category pairs: activity organizer (senior association or sport club), focus (competition or leisure), organization (individual or team) and intensity (low or medium to high).

Here follows information about the categorisation. Activity organizer refers to the organizing club or association. A senior association is one where activities take place only for older adults (60+). Sport clubs can have both senior-specific initiatives and activities for other age groups and different purposes; the participants included in this study are in senior activities only. The activity focus, with the possibility to compete or participate exclusively as a leisure activity. Here activities are categorized as either/or; i.e., if there is a possibility to compete (ex. golf, tennis) even it is categorized as competition, even if someone chooses not to do so. Activity organization concerns activities requiring a team (or at least an opponent to play against) to take part in, as opposed to individually performed activities (though still in a group). Activity intensity describes the level of intensity of the activity and was labelled as low intensity (MET < 4) or medium-to-high intensity (MET > 5) according to the MET table scale (28).

The 27 initiatives in our final sample were relatively evenly distributed according to number of participants between the activity pairs in the categories activity organizer and activity intensity, but differ in activity focus and activity organization. (Activity focus had more leisure than competition initiatives, and activity organization had more individual than team initiatives; see Table 2). Sports and physical activities represented in the 27 initiatives are; group gymnastics, water exercise, which covered almost half of the participants. The other initiatives included are resistance training, tennis, table tennis, indoor boule, walking football, senior Tabata, dancing, golf, bowling, wrestling and outdoor walking. The sports and activities are not seasonally dependent, but ongoing all year round.

**Table 2:** Number of initiatives and participants in the activity pairs.

	Activity organizer		Activity focus		Activity organization		Activity intensity	
	Senior association	Sport club	Competition	Leisure	Individual	Team	Low	Medium to high
*N* = Initiatives	17	10	8	19	17	10	17	10
*n* = Participants (%)	164 (44%)	208 (56%)	111 (30%)	261 (70%)	260 (70%)	112 (30%)	215 (58%)	157 (42%)

The activity categories were then related to the organizational characteristics of sport and physical activity initiatives regarding costs (average in Euros), booking possibilities (three categories), and recruitment to the initiative (three categories). These variables were chosen because they were the quantitative variables on organizational level in SPAHRQ that answered to the first research question. The activity categories were then related to the participants' demographic factors age, gender, education level, city size, and time spent in physical activity per week (overall) to answer to the second research question. Education was assessed and classified according to the International Standard Classification of Education from UNESCO (43) with (1) low-level education (at most nine years of school), (2) medium-level education (secondary school) and (3) high-level education (high school and university studies).

### Statistical analysis

Descriptive statistics, chi-square tests, *t*-tests and multivariate logistic regression analysis were performed to determine differences between independent variables related to the four activity pairs. Covariates in the multivariate logistic regression were participants' age (continuous), gender (men, women), education (categorical), and city size (categorical). The percentage of missing data for the logistic regressions was 8%, and this was left out of the analysis in listwise deletion (removing all data for a case that has one or more missing values) (44). The data analysis was performed using IBM SPSS version 26.

## Results

The study included 27 sport and physical activity initiatives with a total of 372 participants, ranging from 60 to 96 years of age (mean age: 74.4 ± 7 years); 60% were women. Almost all the initiatives had participants with a high level of physical activity in everyday life; 93% met the general criteria of 150 min/week of physical activity from self-rated data obtained in the SPAHRQ questionnaire (mean 634 ± 464 min/week) and a range of 90. Below, the results on the first research question on, characteristics of the sport and PA initiatives are presented. Followed by the results for the second research question, what characterizes the participants in the different initiatives.

### What characterizes the different initiatives?

Costs for the initiatives vary from free (zero) up to around €2,000 for a one-year membership and participation, M (SD) = €149 (117). The lowest costs are found in senior associations, leisure-focused initiatives, individual initiatives, and low-intensity activities (Table 3). In senior associations, the cost per year is on average €46, compared to an average of €217 in sport clubs. The most expensive activities were tennis and golf. There are also differences in how the participants book their activities in the different initiatives. In senior associations, most activities were booked per semester 60%. In sport clubs the most common booking system was per occasion, 40%. In team sports (almost always in sport clubs), the most common way of booking was per semester (46%). The recruitment into the initiatives on the other hand was very similar between the different activity categories. Around 70% were recruited by an internal contacts like family, friends, and colleagues, around 25% by an external contacts like advertisements or senior exhibitions, and around 5% at the participant's own initiative.

**Table 3:** Organizational characteristics compared in the four activity categories (*N* = 27 initiatives, *n* = 343–365).

Activity categories	Activity organizer		Activity focus		Activity organization		Activity intensity	
Organizational variables	Senior association	Sport club	Competition	Leisure	Individual	Team	Low intensive	Medium- to high intensive
Cost (€), Mean (SD)	**€46 (5)**	**€217 (18)**	**€242 (34)**	**€104 (6)**	**€136 (9)**	**€162 (31)**	**€127 (24)**	**€155 (9)**
Booking (*n* = 356)								
Per semester (*n* = 165)	**60%**	**36%**	**41%**	**49%**	**47%**	**45%**	50%	44%
Each occasion (*n* = 108)	**18%**	**40%**	**22%**	**34%**	**38%**	**13%**	25%	34%
Other (*n* = 83)	**22%**	**24%**	**37%**	**17%**	**15%**	**42%**	25%	22%
Recruitment (*n* = 361)								
Internal^a^ (*n* = 260)	68%	75%	71%	74%	71%	75%	69%	74%
External^b^ (*n* = 85)	27%	21%	24%	22%	24%	22%	27%	21%
Own initiative^c^ (*n* = 16)	5%	4%	5%	4%	5%	3%	4%	5%

*p *< 0.05 within each pair, written in bold.

^a^ Through friends, family, relative and partner.

^b^ Through advertisement in newspaper, visiting a lecture or social media.

^c^ Searched for or contacted initiatives themselves.

### What characterizes the participants in the different initiatives?

The initiatives with the most participants were found in group gymnastics and water exercise, which covered almost half of the participants. Sport clubs have significantly more younger participants (age group 60 to79 years) than senior associations (89% vs. 72%, *p* < 0.001) (Table 4). Senior associations further had significantly more female than male participants (71% vs. 50%, *p* < 0.001). Sport clubs had significantly more participants living in cities (74% vs. 30%, *p* < 0.001) than senior associations. Due to the activity focus, significantly more men participated in competitive activities than women (78% vs. 11%, *p* < 0.001). The same result was found for team sports when comparing men and women (60% vs. 10%, *p* < 0.001). Initiatives with medium-to-high intensity level of the PA had more participants in the 60 to 79 age-range than low intensity PA (89% vs. 71%, *p* < 0.001). A majority of initiatives had participants who began with the activity 1-4 years ago.

**Table 4:** The participants in the different initiatives, compared across the four activity categories (*N* = 27 initiatives, *n* = 343–365).

Activity categories	Activity organizer		Activity focus		Activity organization		Activity intensity	
Individual variables	Senior association	Sport club	Competition	Leisure	Individual	Team	Low intensive	Medium- to high intensive
Gender (*n* = 372)								
Male	**29%**	**50%**	**78%**	**24%**	**23%**	**80%**	38%	42%
Female	**71%**	**50%**	**22%**	**76%**	**77%**	**20%**	62%	58%
Age (*n* = 368)								
60–69 years	**12%**	**27%**	22%	20%	20%	21%	**14%**	**25%**
70–74 years	**28%**	**31%**	35%	27%	29%	30%	**26%**	**32%**
75–79 years	**32%**	**31%**	30%	32%	31%	32%	**31%**	**31%**
80–96 years	**28%**	**11%**	13%	21%	20%	17%	**29%**	**11%**
Education (*n* = 370)								
Primary school	**25%**	**45%**	26%	37%	36%	28%	**45%**	**25%**
Secondary school	**27%**	**27%**	27%	27%	26%	31%	**28%**	**26%**
University education	**49%**	**28%**	47%	36%	39%	41%	**27%**	**49%**
City size (*n* = 371)								
City	**30%**	**74%**	**62%**	**52%**	53%	59%	34%	70%
Smaller town	**52%**	**15%**	**20%**	**36%**	34%	25%	50%	18%
Countryside	**18%**	**11%**	**18%**	**12%**	13%	16%	16%	12%
Years spent in initiative (*n* = 345)								
1 month-1 year	**18%**	**13%**	14%	15%	**17%**	**9%**	**22%**	**10%**
1–4 years	**51%**	**43%**	36%	51%	**50%**	**38%**	**42%**	**49%**
5–9 years	**16%**	**13%**	18%	13%	**12%**	**21%**	**16%**	**13%**
10 years or more	**15%**	**31%**	32%	21%	**21%**	**32%**	**20%**	**28%**

*p* < 0.05 within each pair, written in bold.

A multivariate logistic regression was performed to control for all the variables in one block (Table 5). Age and gender were the variables that had the most effect on which initiatives the participants were involved in. The greatest difference and main finding is that men participate in team sports to a greater extent than women (OR = 21.6). Concerning the focus on the activity, younger participants and men participate in competitive sports to a greater extent than women in this study. In activities with medium-to-high intensity, compared to low, the age is higher. The results also reveal that which organization that organizes the activity is significant for who the participants are. In sport clubs, compared to senior associations, we find male and higher-educated participants to a greater extent, and people living in small towns to a lesser extent.

**Table 5:** Multivariate logistic regression with the clustered initiatives as dependent variables adjusted for age, gender, education, city size and active years in the physical activity (*n* = 343). .

Dichotomous variables *n* = 343	Activity organizer	Activity Focus	Activity organization	Activity intensity
OR (95% CI)	Sport clubs compared to Senior associations	Leisure compared to competition	Team compared to individual	Medium to high compared to low
Age (count.)	**1.2** **(1.1–1.3)**	**0.96** **(0.92–0.99)**	1.0 (0.98–1.06)	**1.13** **(1.1–1.2)**
Gender (Ref: Woman)	1.0	1.0	1.0	1.0
* *Men	**2.8** **(1.6–5.2)**	**0.1** **(0.04–0.13)**	**21.6** **(11.0–42.4)**	0.9 (0.5–1.6)
Education (Ref: Primary school)	1.0	1.0	1.0	1.0
Secondary school	**3.5** **(1.8–7.1)**	0.7 (0.4–1.5)	0.8 (0.4–1.7)	**3.8** **(2.0–7.3)**
University education	**2.3** **(1.1–4.6)**	0.5 (0.3–1.01)	1.2 (0.6–2.5)	**2.4** **(1.3–4.7)**
City size (Ref: City)	1.0	1.0	1.0	1.0
Small town	**0.2** **(0.1–0.3)**	1.2 (0.5–2.7)	0.6 (0.3–1.3)	**0.3** **(0.2–0.7)**
Countryside	2.3 (0.98–5.4)	0.6 (0.2–1.4)	0.8 (0.3–1.9)	**2.4** **(1.1–5.3)**
Years spent in initiative				
(Ref. *1 month -1 year)*	1.0	1.0	1.0	1.0
1–4 years (*n* = 159)	**3.3** **(1.3–8.4)**	0.7 (0.3–1.6)	**3.7** **(1.4–9.7)**	**3.4** **(1.4–8.0)**
5–9 years (*n* = 50)	**2.6** **(1.3–5.5)**	0.6 (0.3–1.2)	1.5 (0.7–3.0)	1.1 (0.6–2.2)
10-years and more (*n* = 84)	2.3 (0.9–5.8)	1.7 (0.7–4.1)	0.4 (0.2–1.1)	1.6 (0.7–3.7)

*p *< 0.05, written in bold. OR, Odds ratio. Ref., Reference category.

These findings indicate that the characteristics of both the participants and the initiatives in which they participate are varied.

## Discussion

This study has identified characteristics of sport and physical activity initiatives that older adults in Sweden participate in, in terms of how the initiatives are organized and what characterizes the participants in the different initiatives. Exploring sport and physical activity initiatives that are ongoing and investigating how they are designed are a way to better understand older adults' participation in sports and PA and thus be able to organize activities that are more beneficial for their participants. It is of great importance for society as a whole that older adults are physically active and that there are relevant activities for them to participate in.

Our main findings predominantly concern the differences between different organizers, in this study divided in sport clubs and senior associations. *Who* organizes the sport and physical activity initiative seems to affect the characteristics of *how* it is organized and *what characterizes* the participants in them. Secondly, despite the different characters of sport and physical activity initiatives, the majority of the older adults are recruited by internal contacts like friends and family. Thirdly, which older adults that participate in which sport and physical activity initiatives can be explained mainly by the age and gender of the participants.

Below we will discuss our main findings in relation to previous research and then present our findings in relation to recommendations for possibilities for how older adults can “drop-in”, “drop-through” and “drop-over” in sport and physical activity initiatives as a way to reduce drop-out, in line with the suggestions from Geidne and Quennerstedt (26) and Geidne et al. (25).

### Results discussion

To our knowledge, research comparing different types of sport and PA initiatives for older adults in relation to costs, booking and recruitment is lacking. Consistent with previous studies (45, 46) we show that the sports club and the senior associations creates a valuable setting to promote health, with our research adding fresh insights to the small but growing corpus of evidence specifically on health-promoting initiatives for older adults.

Which actors that run sport and organized physical activity for older adults can differ between countries, but also within countries (47). In Sweden, most sports are organized by sport clubs that are non-governmental and are included under the umbrella organization the Swedish Sports Confederation. Organized PA can be organized by sport clubs, but also by other organizations, both non-governmental and private. In this study, one of our main findings was the significant differences in both the character of organizations and the participants in them, regarding whether it was a sport club or a senior association that organized the activity. Sport clubs were generally more expensive, even if some sport clubs have senior discounts and cheaper booking in daytime. However, if equipment is necessary for an activity, costs can still be high. One explanation may be that senior associations have less expenditures for renting spaces for activities, since several of the initiatives take place in rooms also used for meetings and by other organizations. Senior associations also had more booking per semester than per occasion, as is more common in sport clubs. There was a large difference, however, between different sports, with golf, for example, more often being booked per occasion, but not table tennis.

Another important finding of our study is that, regardless of what characterizes the activity and the different participants, a vast majority of the participants are recruited by family, friends, colleagues or other internal contacts. Similar results are found by (21, 23), who conclude that most older adults are recruited by internal contacts and personal invitations. External recruiting is important, but needs better and more explicit strategies, according to West et al. (48). If the majority of recruiting is internal it is a risk that many older adults will not participate to this kind of sport and PA initiatives also in the future. This can also be discussed in relation to the group of participants included in our study, because they are a very homogeneous group in terms of socio-economic status and health (14). If most participants are recruited through family and friends, there is a risk that some groups never get invited to join or even know that such activities exist, which will affect the diversity of participation and also lead to social inequality (24).

The third main finding that stood out concerned the differences related to age and gender. Men were more than 21 times more likely than women to be participants in team sports. One explanation of this could be that women who were young in the 1960–70s often did not have the same opportunities to participate in team sports, and what you do early in life often affects what you do later (12). On the other hand, women participated more often in individual activities. The gender differences in team sport at an older age are in some studies explained by women reported having more barriers to exercise than men (49). Another study also strengthens the argumentation that males were more motivated than females by mastery and competition in sports, whereas females were motivated more than males by appearance and physical condition (50). The results also show that the intensity of the activity decreased with increasing age. This could also be related to the fact that older women more often participate in senior associations, where individual low-intensity activities are more common. In senior associations, the cost of the activities was also lower and, as Wong et al. (51) also state, the financial insecurity that some groups experience in later life, for example women, can impact their ability to afford and access physical activities. Due to the results that men often participated in competitive sports than women are in line with what both Hirvensalo and Lintunen (52) and Chen et al. (53) found. Playing competitive sport at younger age was a significant predictor of sport involvement during old adulthood (53).

### Recommendations for drop-in, drop-through and drop over in relation to older adults

In response to the lack of explicit recommendations for how organizers could organize activities to suit as many people as possible for as long as possible, in this section we will offer some suggestions regarding how older adults can drop-in, drop-through and drop-over in sport and physical activity initiatives as a way to reduce drop-out.

*Drop-in* concerns recommendations for how to lower the threshold for older adults to start to be active, from the beginning, after a break, or when switching to a new activity. For older adults who used to participate in sports (or have done so continuously since they were younger) the threshold to continuing when older or starting a new activity is probably lower than for someone who has never participated in sports. On the other hand, the threshold to start doing a physical activity in a senior association can be easier to overcome than in other organizations. Here different types of recruitment strategies will be needed to recruit different target groups. As West et al. (21) discuss, other forms of marketing could be a strategy for recruiting and finding new target groups. However, as Wong et al. (51) state, it is not just marketing strategies that are needed; there is also a need for resources for local organizations directly promoting older adults' participation in sport and physical activity.

Another recommendation for lowering the threshold to drop-in is to design different forms of sport and physical activity for the quite heterogeneous group of older adults, not only when it comes to which sport, but also focus, organization and booking opportunities. How an activity is booked could make it more or less easy for different people to drop in to an activity. Finding different recruitment strategies could then potentially increase diversity and reduce social inequality in sport and physical activity (24).

A third recommendation related to drop-in is to start with short-term projects as a springboard into ongoing activities. The focus in this study was on the ongoing activities, but to increase drop-in for specific groups, or when testing a new form, short-term initiatives can be a complement. It is then important to make a short-term initiative sustainable, for example, by cooperating with other actors, such as outdoor associations, the scouts, and pensioners organizations (drop-over), or having a strategy for keeping participants within the organization (drop-through).

*Drop-through* concerns recommendations for how to lower the threshold for older adults to change activities within an organization. One of our main findings in this study is that age is significant for which activity older adults participate in. Hence, there should be an opportunity for older adults (and others) to change their form of activity within the same organization, for example from medium-high intensity to low, using a golf cart but still playing golf, or doing walking football instead of football. Changing the form of sport and physical activity can be done while maintaining the social relations that older adults have built up and which most of them find to be a meaningful aspect of participating (14).

Here, a life-course perspective on how to organize sports is recommended (23, 54, 55). In line with our findings that older adults often reveal that they have been members for a long time and were recruited by internal contacts, such as when their children started or even earlier, planning for drop-through could be a successful strategy. In these features we see possibilities for the organizations to also work with external recruiting in the future, with the aim of reaching a broader public that is less familiar with the settings of sport clubs or physical activity initiatives.

*Drop-over* concerns recommendations for how to lower the threshold for older adults to change from one organization to another. Cooperation between different organizations, for example between a summer and a winter sport, or a senior association and a sport club, could be very beneficial for older adults. Cooperation with other senior associations (also for health care) is also emphasized as an important way of recruiting and communicating in other studies (21, 23, 24). Geographical considerations, like living in the countryside or in a city, can further affect whether the need for a drop-over alternative is even greater than for drop-through, depending on the size of the organization or the number of organizations in the neighbourhood. It is important to take a holistic approach to the question of increasing drop-in and reducing drop-out among the older adult members.

### Limitations and strengths

We have, of course, some reflections on the choices we have made during this journey. For this study we developed a new questionnaire, the SPAHRQ, which of course may have some teething pains. One limitation is that we did not have a question on how long the respondents had been active participants in any sport; we only asked about their current activity. It would have been interesting to compare those. On the other hand, the SPAHRQ was developed from an in-depth theoretical framework for asking questions in a certain way to convey salutogenic theory (29), which contributes to deepening the knowledge about both organizational and individual factors and the relation between them.

We also developed the organizational category pairs of organizer, focus, organization and intensity, inspired by Eime et al. (56) mode, type and setting. In the future it would be interesting if more categories could be developed to explore different characteristics of sport and physical activity initiatives, both for older adults and others, as well as in different contexts. Another sample, which also included for example private gyms with organized physical activity, might have further deepened our results, and this could also be developed in future studies.

Another limitation may be the self-rated data. In some studies, participants tend to overrate time spent in physical activity per week (57). This is a minor problem, however, in this type of study, where the aim of this variable is mostly to describe who the participants are. In this study it also does not matter that the participants are a quite homogeneous group, because the focus is on ongoing initiatives and how these could be organized, and the participants in them are the way they are. Of course, we know that these are very healthy older adults with good preconditions to participate in sport and physical activity.

### Conclusion

Individual-level recommendations for how much physical activity people in different ages should perform to gain health benefits are not enough to get older adults to participate in different sport and PA initiatives. In this study, we have proposed some recommendations for how organizations could organize activities to suit as many people as possible for as long as possible.

When considering recommendations like these, with a specific focus on older adults, we conclude that older adults want similar outcomes when participating in sport and physical activity, but that they seek this in different ways. To attract as many older adults as possible, organizations should accordingly work with lowering the threshold *into* sports and physical activity, as well as *within* and *between* different sports and physical activities, in order to raise the threshold to dropping out of sport and physical activity (58, 59).

## Data Availability

The datasets presented in this article are not readily available because https://doi.org/10.5878/h8qw-ax80. Requests to access the datasets should be directed to helena.ericson@oru.se.

## References

[1] Chodzko-Zajko WJ Proctor DN Singh MAF Minson CT Nigg CR Salem GJ Exercise and physical activity for older adults. Med Sci Sports Exerc. (2009) 41:1510–30. 10.1249/MSS.0b013e3181a0c95c 19516148

[2] Lera-Lopez F Marco R. Sports participation, physical activity, and health in the European regions. J Sports Sci. (2018) 36:1784–91. 10.1080/02640414.2017.1418810 29272203

[3] Smith GL Banting L Eime R O’Sullivan G Van uffelen JGZ. The association between social support and physical activity in older adults: a systematic review. Int J Behav Nutr Phys Act. (2017) 14:1–21. 10.1186/s12966-017-0613-9 28449673 PMC5408452

[4] Westerbeek H Eime R. The physical activity and sport participation framework-A policy model toward being physically active across the lifespan. Front Sports Active Living. (2021) 3:90. 10.3389/fspor.2021.608593 PMC8138121 34027402

[5] Rhodes RE Janssen I Bredin SSD Warburton DER Bauman A. Physical activity: health impact, prevalence, correlates and interventions. Psychol Health. (2017) 32:942–75. 10.1080/08870446.2017.1325486 28554222

[6] World Health Organization (WHO). World report on ageing and health. Geneva: World Health Organization (2015).

[7] Eime RM Harvey JT Charity MJ Casey MM Westerbeek H Payne WR. Age profiles of sport participants. BMC Sports Sci Med Rehabil. (2016) 8:6. 10.1186/s13102-016-0031-3 26973792 PMC4788892

[8] Jenkin CR Eime RM Westerbeek H Van Uffelen JGZ. Sport for adults aged 50+ years: participation benefits and barriers. J Aging Phys Act. (2018) 26:363–71. 10.1123/japa.2017-0092 28952860

[9] Eurobarometer. Standard Eurobarometer 89 Spring 2018. *European Commission* (2018).

[10] Jenkin CR Eime RM Westerbeek H O’Sullivan G Van Uffelen JGZ. Sport and ageing: a systematic review of the determinants and trends of participation in sport for older adults. BMC Public Health. (2017) 17:976. 10.1186/s12889-017-4970-8 29273036 PMC5741887

[11] Amireault S Baier JM Spencer JR. Physical activity preferences among older adults: a systematic review. J Aging Phys Act. (2018) 27:128–39. 10.1123/japa.2017-0234 29283793

[12] Dionigi RA Fraser-Thomas J Stone RC Gayman AM. Psychosocial development through masters sport: what can be gained from youth sport models? J Sports Sci. (2018) 36:1533–41. 10.1080/02640414.2017.1400147 29106335

[13] Huffman MK Amireault S. What keeps them going, and what gets them back? Older adults’ beliefs about physical activity maintenance. Gerontologist. (2021) 61:392–402. 10.1093/geront/gnaa087 32622348

[14] Ericson H Quennerstedt M Geidne S. Physical activity as a health resource: a cross-sectional survey applying a salutogenic approach to what older adults consider meaningful in organised physical activity initiatives. Health Psychol Behav Med. (2021) 9:858–74. 10.1080/21642850.2021.1986400 34650835 PMC8510608

[15] Mcgowan LJ Devereux-Fitzgerald A Powell R French DP. How acceptable do older adults find the concept of being physically active? A systematic review and meta-synthesis. Int Rev Sport Exerc Psychol. (2018) 11:1–24. 10.1080/1750984X.2016.1272705

[16] Koster A Stenholm S Schrack JA. The benefits of physical activity for older people. In: Nyman SR, Barker A, Haines T, Horton K, Musselwhite C, Peeters G, et al. editors. The Palgrave handbook of ageing and physical activity promotion. Cham: Palgrave Macmillan (2018). p. 43–60. 10.1007/978-3-319-71291-8.

[17] Oppezzo M Wegner L Gross JJ Schwartz DL Eckley T King AC What moves you? Physical activity strategies in older women. J Health Psychol. (2021) 27(9):2027–40. 10.1177/13591053211014593. 34006131 PMC9059481

[18] Hoare E Stavreski B Jennings GL Kingwell BA. Exploring motivation and barriers to physical activity among active and inactive Australian adults. Sports. (2017) 5:47. 10.3390/sports5030047 29910407 PMC5968958

[19] Jenkin C Eime R Van Uffelen J Westerbeek H. How to re-engage older adults in community sport? Reasons for drop-out and re-engagement. Leisure Stud. (2021) 40(4):441–53. 10.1080/02614367.2021.1888310

[20] Jenkin C Van Uffelen JGZ O’Sullivan G Harvey J Eime RM Westerbeek H. Marketing up the wrong tree? Organisational perspectives on attracting and/or retaining older adults in sport. Front Sports Act Living. (2021) 3:772361. 10.3389/fspor.2021.772361 34901849 PMC8662314

[21] West S Naar JJ Son JS Liechty T. Promoting team sport participation among older women. J Park Recreat Admi. (2019) 37(4):33–50. 10.18666/JPRA-2019-9118.

[22] Corepal R Zhang JY Grover S Hubball H Ashe MC. Walking soccer: a systematic review of a modified sport. Scand J Med Sci Sports. (2020) 30:2282–90. 10.1111/sms.13772 32671884

[23] Naar JJ Wong JD West ST Son JS Liechty T. A socioecological approach to women’s participation in competitive softball during middle and late adulthood. Top Geriatr Rehabil. (2017) 33:170–81. 10.1097/TGR.0000000000000153

[24] Wolter V Dohle M Sobo L. Physical activities for older adults: are local co-operations of sports clubs and care partners an option to increase access? German J Exerc Sport Res. (2021) 51:468–73. 10.1007/s12662-021-00761-3

[25] Geidne S Ericson H Quennerstedt M. De äldre och föreningsidrotten. Riksidrottsförbundet. (2022) 2022:4. ISBN: 978-91-87385-37-7.

[26] Geidne S Quennerstedt M. Youth perspectives on what makes a sports club a health-promoting setting-viewed through a salutogenic settings-based Lens. Int J Environ Res Public Health. (2021) 18:7704. 10.3390/ijerph18147704 34300153 PMC8307637

[27] Wagnsson S Gustafsson H Libäck J Podlog LW. Lessons learned from a multi-level intervention program to reduce Swedish female Floorballers’ dropout rate. J Sport Psychol Action. (2021) 12:226–44. 10.1080/21520704.2020.1850576

[28] Ainsworth BE Haskell WL Whitt MC Irwin ML Swartz AM Strath SJ Compendium of physical activities: an update of activity codes and MET intensities. Med Sci Sports Exercise. (2000) 32:S498–504. 10.1097/00005768-200009001-00009 10993420

[29] Antonovsky A. Health, stress, and coping. New Perspect Mental Phys Well-Being. (1979):12–37. ISBN: 0875894127.

[30] Antonovsky A. The salutogenic model as a theory to guide health promotion. Health Promot Int. (1996) 11:11–8. 10.1093/heapro/11.1.11

[31] Mccuaig L Quennerstedt M. Health by stealth—exploring the sociocultural dimensions of salutogenesis for sport, health and physical education research. Sport Educ Soc. (2018) 23:111–22. 10.1080/13573322.2016.1151779

[32] Geidne S Quennerstedt M Eriksson C. The youth sports club as a health-promoting setting: an integrative review of research. Scand J Public Health. (2013) 41:269–83. 10.1177/1403494812473204 23349167 PMC3807854

[33] Van hoye A Johnson S Geidne S Donaldson A Rostan F Lemonnier F The health promoting sports club model: an intervention planning framework. Health Promot Int. (2021) 36:811–23. 10.1093/heapro/daaa093 33111939

[34] Peterson T. Föreningsfostran och tävlingsfostran. En utvärdering av statens stöd till idrotten, SOU 2008: 59 [Club fosterage and competition fosterage. An evaluation of government support to the Swedish sport movement, SOU 2008: 59]. Stockholm: Fritze. Fritze Stockholm (2008).

[35] Bäckström Å Book K Carlsson B Fahlström PG. Sport management. Del 1: Idrottens organisationer i en svensk kontext. SISU idrottsböcker (2018).

[36] Bjärsholm D. Networking as a cornerstone within the practice of social entrepreneurship in sport. Eur Sport Manage Q. (2019) 19:120–37. 10.1080/16184742.2018.1546753

[37] Etikan I Alkassim R Abubakar S. Comparision of snowball sampling and sequential sampling technique. Biometrics Biostatistics Int J. (2016) 3:55. 10.15406/bbij.2015.03.00055

[38] Ericson H Quennerstedt M Skoog T Johansson M. Health resources, ageing and physical activity: a study of physically active women aged 69-75 years. Q Res Sport Exerc Health. (2018) 10:206–22. 10.1080/2159676X.2017.1393453

[39] Antonovsky A. Unraveling the mystery of health: How people manage stress and stay well. Stockholm: Jossey-bass (1987).

[40] Super S Verkooijen K Koelen M. The role of community sports coaches in creating optimal social conditions for life skill development and transferability–a salutogenic perspective. Sport Educ Soc. (2018) 23:173–85. 10.1080/13573322.2016.1145109

[41] Mittelmark MB Bauer GF Vaandrager L Pelikan JM Sagy S Eriksson M The handbook of salutogenesis. Basel: Springer Nature (2022). 36121968

[42] Eime RM Harvey JT Sawyer NA Craike MJ Symons CM Polman RC Understanding the contexts of adolescent female participation in sport and physical activity. Res Q Exerc Sport. (2013) 84:157–66. 10.1080/02701367.2013.784846 23930541

[43] Isced U. International standard classification of education 2011. Bingley: UNESCO Institute for Statistics Montreal (2012).

[44] Pallant J. SPSS Survival manual: a step by step guide to data analysis using IBM SPSS. New York, NY: Routledge (2020).

[45] Costello L McDermott ML Patel P Dare J. “A lot better than medicine’-self-organised ocean swimming groups as facilitators for healthy ageing. Health Place. (2019) 60:102212. 10.1016/j.healthplace.2019.102212 31610442

[46] Jackman P Lane A Allen-Collinson J Henderson H. Older adults’ and service providers’ experiences of a settings-based health promotion initiative in English football. Health Promot Int. (2023) 38(3):1–12. 10.1093/heapro/daad027 PMC10797665 37339012

[47] Swedish Sport Confederation. Idrotten vill: idrottsrörelsens idéprogram:[antagen av RF-stämman 2009]. Stockholm: Riksidrottsförbundet (2009).

[48] West S Naar JJ Son JS Liechty T. Promoting team sport participation among older women. J Park Recreat Admi. (2019b) 37:35–50. 10.18666/JPRA-2019-9118.

[49] Hickey ME Mason SE. Age and gender differences in particpation rates, motivators for, and barriers to exercise. Mod Psychol Stud. (2017) 22(2):3. htps://scholar.utc.edu/mps/vol22/iss2/3

[50] Molanorouzi K Khoo S Morris T. Motives for adult participation in physical activity: type of activity, age, and gender. BMC Public Health. (2015) 15(1):1–12. 10.1186/s12889-015-1429-7 25637384 PMC4314738

[51] Wong Son JD West JS Naar ST Liechty JJT. A life course examination of women’s team sport participation in late adulthood. J Aging Phys Act. (2018) 27:73–82. 10.1123/japa.2017-0193 29722593

[52] Hirvensalo M Lintunen T. Life-course perspective for physical activity and sports participation. Eur Rev Aging Phys Act. (2011) 8(1):13–22. 10.1007/s11556-010-0076-3

[53] Chen G Janke MC Liechty T Wong JD West ST Son JS Sport participation for adults aged 50+ years: a socioecological analysis. Int J Aging Human Dev. (2022). 97(3):354–73. 10.1177/00914150221143958 36464642

[54] Sansano-Nadal O Giné-Garriga M Brach JS Wert DM Jerez-Roig J Guerra-Balic M Exercise-based interventions to enhance long-term sustainability of physical activity in older adults: a systematic review and meta-analysis of randomized clinical trials. Int J Environ Res Public Health. (2019) 16:2527. 10.3390/ijerph16142527 31311165 PMC6678490

[55] Borgers J Vanreusel B Lefevre J Scheerder J. Involvement in non-club organized sport: organizational patterns of sport participation from a longitudinal life course perspective. Eur J Sport Soc. (2018) 15:58–77. 10.1080/16138171.2018.1438079

[56] Eime RM Harvey JT Charity MJ Nelson R. Demographic characteristics and type/frequency of physical activity participation in a large sample of 21,603 Australian people. BMC Public Health. (2018) 18:1–10. 10.1186/s12889-017-4524-0 PMC5989390 29871601

[57] Downs A Van Hoomissen J Lafrenz A Julka DL. Accelerometer-measured versus self-reported physical activity in college students: implications for research and practice. J Am Coll Health. (2014) 62(3):204–12. 10.1080/07448481.2013.877018 24377672

[58] Lindström B Eriksson M. Salutogenesis. J Epidemiol Community Health. (2005) 59(6):440–2. 10.1136/jech.2005.034777 15911636 PMC1757059

[59] Pinheiro MB Howard K Oliveira JS Kwok WS Tiedemann A Wang B Cost-effectiveness of physical activity programs and services for older adults: a scoping review. Age Ageing. (2023) 52(3). 10.1093/ageing/afad023 PMC10024893 36934340

